# Cannabinoids: an Effective Treatment for Chemotherapy-Induced Peripheral Neurotoxicity?

**DOI:** 10.1007/s13311-021-01127-1

**Published:** 2021-10-19

**Authors:** Guido Cavaletti, Paola Marmiroli, Cynthia L. Renn, Susan G. Dorsey, Maria Pina Serra, Marina Quartu, Cristina Meregalli

**Affiliations:** 1 Experimental Neurology Unit, School of Medicine and Surgery, University of Milano Bicocca, via Cadore 48, Monza, Italy; 2 Department of Biotechnology and Biosciences, University of Milano-Bicocca, Piazza della Scienza, 2, Milano, Italy; 3 Milan Center for Neuroscience, University of Milano Bicocca, Piazza Ateneo Nuovo 1, Milano, Italy; 4 Department of Pain and Translational Science, School of Nursing, University of Maryland, 655 West Lombard Street, Baltimore, MD 21201 USA; 5 Department of Biomedical Sciences, University of Cagliari, Cittadella Universitaria, 09042 Monserrato, Italy

**Keywords:** Chemotherapy, Neuropathy, Endocannabinoid system, Cannabinoid receptors, Treatment

## Abstract

Chemotherapy-induced peripheral neurotoxicity (CIPN) is one of the most frequent side effects of antineoplastic treatment, particularly of lung, breast, prostate, gastrointestinal, and germinal cancers, as well as of different forms of leukemia, lymphoma, and multiple myeloma. Currently, no effective therapies are available for CIPN prevention, and symptomatic treatment is frequently ineffective; thus, several clinical trials are addressing this unmet clinical need. Among possible pharmacological treatments of CIPN, modulation of the endocannabinoid system might be particularly promising, especially in those CIPN types where analgesia and neuroinflammation modulation might be beneficial. In fact, several clinical trials are ongoing with the specific aim to better investigate the changes in endocannabinoid levels induced by systemic chemotherapy and the possible role of endocannabinoid system modulation to provide relief from CIPN symptoms, a hypothesis supported by preclinical evidence but never consistently demonstrated in patients. Interestingly, endocannabinoid system modulation might be one of the mechanisms at the basis of the reported efficacy of exercise and physical therapy in CIPN patients. This possible virtuous interplay will be discussed in this review.

**Supplementary Information:**

The online version contains supplementary material available at 10.1007/s13311-021-01127-1.

## Introduction

Chemotherapy-induced peripheral neurotoxicity (CIPN) is one of the most frequent side effects of the pharmacological treatment of solid and hematological tumors [1]. Although with remarkably different severity and regional differences, CIPN can affect the vast majority of lung, breast, prostate, gastrointestinal, and germinal cancer patients, as well as subjects affected by different forms of leukemia, lymphoma and multiple myeloma, accounting for approximately 75% of all cancer patients.

Besides the impact of CIPN symptoms during chemotherapy, a significant proportion of cancer patients experience long term, or even permanent, persistence of neurotoxicity symptoms and signs, which negatively affects their working and social activities and overall quality of life. No preventive or symptomatic treatment for CIPN has been identified [2, 3]. The recent American Society for Clinical Oncology (ASCO) clinical practice guidelines on prevention and management of CIPN in survivors of adult cancers [4] as well as the European Society for Medical Oncology (ESMO)–European Oncology Nursing Society (EONS)–European Association of Neuro-Oncology (EANO) clinical practice guidelines for diagnosis, prevention, treatment, and follow-up of chemotherapy-induced neurotoxicity [1] provide only moderate recommendation for the use of duloxetine as a symptomatic treatment.

The spectrum of CIPN clinical features is highly variable depending on the different chemotherapeutic drugs, ranging from a nearly pure sensory neuropathy following platinum drugs (e.g., cisplatin, oxaliplatin), thalidomide or proteasome inhibitors (namely bortezomib) to sensorimotor neuropathy induced by taxanes (e.g., paclitaxel, docetaxel), eribulin, epothilones, or vinca alkaloids (remarkably, during vincristine administration autonomic failure might also occur and be dose-limiting). Neuropathic pain is frequent and may be severe in bortezomib-treated patients, while “paclitaxel associated painful syndrome” is also frequent, but less severe and rapidly disappearing, and, hours after oxaliplatin administration cold-induced paresthesias, cramps, and a spectrum of transient symptoms due to nerve hyperexcitability are nearly universal [5, 6].

The marked variability in CIPN clinical features, as well as their different duration, are likely due to different pathogenic events induced in the peripheral nervous system by the various classes of neurotoxic drugs. To date, the knowledge of these underlying mechanisms is largely incomplete; although, emerging evidence points to mitochondrial damage and cellular energy failure with oxidative stress, impaired axonal transport due to tubulin damage, membrane transporter and ion channel interactions, and neuroinflammation as putative relevant events [7–9]. The incomplete knowledge of CIPN pathogenesis is a key obstacle in the design of rationale-based clinical trials aimed at the identification of effective treatments for CIPN.

## Ongoing and Planned Clinical Trials in CIPN

Several clinical trials have attempted to resolve the ongoing clinical needs represented by the prevention or treatment of this potentially dose-limiting side effect of the medical treatment of cancers. Most of the current trials (either ongoing or planned) are registered at the ClinicalTrials.gov website (https://clinicaltrials.gov/), and their analysis provides an unbiased and updated view of the emerging concepts in this clinically relevant field.

At the time that the data were extracted from the ClinicalTrials.gov database (April 30, 2021), 63 were either recruiting or active but not yet recruiting. The clinical trials that were examined were retrieved using “chemotherapy-induced peripheral neuropathy” and “treatment” as the database search terms. After individual review of the trials, 11 of them were excluded from the analysis because they dealt with CIPN monitoring, diagnosis, or biomarker identification rather than treatment. From the remaining 52 clinical trials, several different therapeutic strategies emerged, in some cases re-challenging previously investigated hypotheses and drugs (e.g., duloxetine, lorcaserin, lidocaine), testing new investigational agents (e.g., TRK-750, ART-123), and exploring treatments that currently still lack a confirmed rationale (e.g., cryotherapy with or without limbs compression, scrambler therapy, acupuncture, neuromodulation). However, two groups of studies investigate potentially promising, although until now insufficiently explored, fields. These two groups comprise clinical trials exploring the effects of physical therapeutic approaches (e.g., strength training and other types of exercise) and those examining the efficacy of the modulation of the cannabinoid system (Table 1).

**Table 1:** Summary of the clinical trials for the prevention and/or treatment of chemotherapy-induced peripheral neurotoxicity with an active (either recruiting or not yet recruiting) status registered at ClinicalTrials.gov

Clinical trial description, as reported in ClinicalTrial.gov	Status
Pharmacological treatments (other than cannabinoids, *n* = 16)	
Study of nicotine for pain associated with chemotherapy-induced peripheral neuropathy	Recruiting
Memantine XR and pregabalin for chemotherapy-induced peripheral neuropathy	Recruiting
Comparing lorcaserin versus duloxetine for the treatment of chemotherapy-induced peripheral neuropathy	Not yet recruiting
Botulinum toxin A for the treatment of chemotherapy induced peripheral neuropathy	Recruiting
Integrative medicine for chemotherapy-induced peripheral neuropathy	Recruiting
Duloxetine and neurofeedback training for the treatment of chemotherapy induced peripheral neuropathy	Recruiting
A study to investigate the safety and efficacy of TRK-750 for the treatment of patients with CIPN (Chopin Study)	Not yet recruiting
Effects of a glucoside- and rutinoside-rich material in chemotherapy-induced peripheral neuropathy and related symptoms	Recruiting
Menthol In Neuropathy Trial	Recruiting
Lorcaserin in treating chemotherapy-induced peripheral neuropathy in patients with stage I-IV gastrointestinal or breast cancer	Not yet recruiting
Fingolimod in treating patients with chemotherapy-induced neuropathy	Recruiting
NIAGEN and persistent chemotherapy-induced peripheral neuropathy	Recruiting
A trial measuring ART-123 ability to prevent sensory neuropathy in unresectable mCRC subjects w/oxaliplatin-based chemo	Not yet recruiting
Dextromethorphan in chemotherapy-induced peripheral neuropathy management	Recruiting
Lidocaine for oxaliplatin-induced neuropathy	Active, not recruiting
High dose inorganic selenium for preventing chemotherapy induced peripheral neuropathy	Recruiting
Acupuncture and neuro-modulation (*n* = 10)	
Acupuncture in reducing chemotherapy-induced peripheral neuropathy in participants with stage I-III breast cancer	Active, not recruiting
Acupuncture for peripheral neuropathy induced by paclitaxel in early stage breast cancer	Recruiting
Acupuncture for symptoms of nerve damage	Active, not recruiting
Acupuncture to reduce chemotherapy-induced peripheral neuropathy severity during neoadjuvant or adjuvant weekly paclitaxel chemotherapy in breast cancer patients	Active, not recruiting
Efficacy of acupuncture on chemotherapy-induced peripheral neuropathy (CIPN)-CMUH	Not yet recruiting
Evaluation of the efficacy of acupuncture in chemotherapy induced peripheral neuropathy	Recruiting
Home-based neurofeedback program in treating participants with chemotherapy-induced peripheral neuropathy	Not yet recruiting
Yoga for painful chemotherapy-induced peripheral neuropathy: a pilot, randomized-controlled study	Recruiting
A mind–body intervention for chemotherapy-induced peripheral neuropathy	Active, not recruiting
Effects of neurofeedback on neural function, neuromodulation, and chemotherapy-induced neuropathic pain	Active, not recruiting
Electrical and physical treatments (*n* = 14)	
Ozone therapy in chemotherapy-induced peripheral neuropathy: RCT (O3NPIQ)	Recruiting
PBMT for the prevention of CIPN	Recruiting
MC5-A scrambler therapy or TENS therapy in treating patients with chemotherapy-induced peripheral neuropathy	Active, not recruiting
Scrambler therapy for the reduction of chemotherapy- induced neuropathic pain	Recruiting
Neuromodulation as a treatment for chemotherapy-induced peripheral neuropathy	Not yet recruiting
Testing the effects of transcutaneous electrical nerve stimulation (TENS) on chemotherapy-induced peripheral neuropathy (CIPN)	Recruiting
Spinal cord stimulation in chemotherapy induced neuropathy	Recruiting
Project relief: developing brain stimulation as a treatment for chronic pain	Recruiting
Voxx Human Performance Technology Socks for chemotherapy-induced peripheral neuropathy	Recruiting
The CONTRoL Trial: Cryotherapy vs. cOmpression Neuropathy TRiaL	Recruiting
Cryocompression therapy for peripheral neuropathy in patients with multiple myeloma	Recruiting
Cryocompression to reduce chemotherapy-induced peripheral neuropathy cancer	Not yet recruiting
Oral cryotherapy plus acupressure and acupuncture versus oral cryotherapy for decreasing chemotherapy-induced peripheral neuropathy from oxaliplatin-based chemotherapy in patients with gastrointestinal cancer	Not yet recruiting
Cryotherapy to prevent taxane-induced sensory neuropathy of the hands and feet	Recruiting
Breast/evaluation of cryotherapy and TRPA1 receptors in chemotherapy induced neuropathy	Recruiting
Exercise, rehabilitation and nutrition interventions (*n* = 7)	
Preventing chemotherapy-induced peripheral neuropathy using PRESIONA exercise program	Not yet recruiting
Massage therapy in reducing chemotherapy-induced peripheral neuropathy in patients with gastrointestinal or breast malignancies	Active, not recruiting
Chemotherapy induced peripheral neuropathy (CIPN)	Recruiting
Whole body vibration for the improvement of health and functioning in participants with chemotherapy-induced peripheral neuropathy	Recruiting
Daily hand-held vibration therapy	Recruiting
Exercise and nutrition interventions during chemotherapy K07	Recruiting
Home-based physical activity intervention for taxane-induced CIPN	Recruiting
Cannabinoids (*n* = 4)	
**Cannabidiol for prevention of chemotherapy-induced peripheral neuropathy** (trial planned to be completed in February 2023) Inclusion criteria are the presence of breast or gastrointestinal cancers to be treated with paclitaxel or oxaliplatin, life expectancy ≥ 6 months and Eastern Cooperative Oncology Group (ECOG) performance status ≤ 1 (i.e., restricted in physically strenuous activity but ambulatory and able to carry out work of a light or sedentary nature, e.g., light housework, office work)	Not yet recruiting
**The kinetics of endocannabinoids in patients with chemotherapy induced peripheral neuropathy by using medical cannabis** (trial planned to be completed in April 2023) Patients are eligible if they are scheduled to undergo at least 6 courses of paclitaxel- or 4 courses of oxaliplatin-based chemotherapy	Not yet recruiting
**Cannabinoids for taxane induced peripheral neuropathy** (trial planned to be completed in February 2022) Patients are eligible if they developed following paclitaxel- or docetaxel-based chemotherapy for breast cancer	Recruiting
**Effect of hemp-CBD on patients with CIPN** (trial planned to be completed in April 2022) Patients with non-metastatic breast, colorectal, uterine and ovarian cancer patients who received neoadjuvant or adjuvant therapy that included taxanes or oxaliplatin are eligible	Recruiting

## The Endocannabinoid System

A detailed description of the endocannabinoid system is beyond the scope of this review, but some basic concepts may be helpful to better understand the rationale supporting their use in CIPN patients. The endocannabinoid system is fundamental in the development of the nervous system, as well as in the mature nervous system where it modulates network function and neuronal activity [10]. As a whole, the system includes endogenous cannabinoids of which the best known are arachidonoylethanolamine (also known as anandamide, AEA) and 2-arachidonoylglycerol (2-AG), cannabinoid receptors, and the proteins that transport, synthesize, and degrade these receptors. More recently, another group of lipids are considered to be endocannabinoids, including the fatty acid ethanolamides, the fatty acid primary amides and the monoacylglycerol-related molecules. Finally, it has been shown that the hemopressin peptide family, derived from α and β chains of hemoglobins, is likely to be a new family of cannabinoids [11]. The endocannabinoid system is highly integrated in the nervous system circuitry and it influences, and is influenced by, many other signaling pathways. Regarding the effects of the drugs acting on the endocannabinoid system, most of the psychoactive effects classically associated with cannabis are mediated through the interaction of Δ9-tetrahydrocannabinol (THC), the major psychotropic constituent of cannabis, with cannabinoid receptors. Cannabidiol (CBD) is another constituent of cannabis, present at variable levels, which interacts with the endocannabinoid system as well as other neuromodulatory systems. Although several non-canonical cannabinoid receptors have been described [11], CB1 cannabinoid receptors (CB1R) and CB2 cannabinoid receptors (CB2R) are the best-characterized cannabinoid receptors. Both are G protein–coupled receptors able to inhibit adenylyl cyclase and certain voltage-sensitive calcium channels, to stimulate mitogen-activated protein (MAP) kinases, and recruit beta-arrestins, among other actions [12, 13]. The diversity of CB1R signaling in different central nervous system (CNS) regions is enhanced by their propensity to heterodimerize with other G protein–coupled receptors, including dopamine D2, opioid receptors, and hypocretin [14]. Moderate to high expression of CB1R has been observed in the cerebral cortex, basal ganglia, amygdala, hypothalamus, periaqueductal gray, brainstem medullary nuclei (such as the nucleus of the solitary tract and area postrema), and cerebellum. Moderate CB1R expression has also been found in the spinal cord (dorsal horn and lamina I, III, and X), with dense CB1R-positive fibers identified in the ventral horn. Their location in the periaqueductal grey matter and spinal cord dorsal horn (SCDH) may explain their involvement in pain sensation and modulation. Apart from the CNS, CB1R expression was reported in the somatic and autonomic peripheral nervous systems [15].

In the adult CNS, CB1R are most abundant on specific populations of GABAergic interneurons [16], but they are also present on a wide range of glutamatergic, cholinergic, glycinergic, and serotonergic neurons [17]. Their major role in modulating synaptic transmission is reflected by their predominant localization on synaptic terminals [18]. However, CB1R are not restricted to neurons, since they are also expressed by some astrocytes [19]. Their expression and role in other glial cells has not yet been confirmed. CB1R are not restricted to the nervous system, but they are also expressed in the skin, liver, muscle, heart, pancreas, lung, reproductive organs, and adipose tissue [15]. By contrast, although they have a neuronal expression [20], CB2R are primarily expressed in cells of immune origin [21, 22], including microglia [20, 23], but also in pancreatic acinar cells, adipocytes, skeletal muscle cells, cardiomyocytes, and endothelial cells [15]. CB2R are also expressed in astrocytes, oligodendrocytes, neural stem/progenitor cells, vascular elements in the brain [11], and are upregulated in the CNS and dorsal root ganglia (DRG) by pathological pain states [24].

## Modulation of the Endocannabinoid System in CIPN

The role of the endocannabinoid system in CIPN has been extensively explored in preclinical animal models, where the modulation of this system has potent anti-nociceptive effects [25–31]. In animal models, cannabinoids suppress neuropathic pain induced by traumatic nerve injury, toxic insults, and metabolic changes [32], although the role of cannabinoid receptors in this process is still not completely known. In fact, while the relevance of the activation of CB1R on DRG neurons to explain the anti-nociceptive effects of cannabinoids has been demonstrated by site-specific drug administration and by tissue-selective knockout [33, 34], the primary site of CB2R-mediated antiallodynic effects is still unclear [35, 36].

### Analgesic Effects of Cannabinoids

Although we did not perform a systematic review of this specific aspect, it might be useful to analyze some results obtained in animal studies. Mulpuri et al. tested 4-{2-[-(1E)-1[(4-propylnaphthalen-1-yl)methylidene]-1H-inden-3-yl]ethyl}morpholine (PrNMI), a compound from a series of synthetic peripherally restricted cannabinoids, in a rat model of cisplatin-induced peripheral neuropathy. In their study, the authors showed that local or systemic administration of PrNMI dose-dependently suppressed CIPN mechanical and cold allodynia without any CNS side effects. In order to investigate the mechanism of action of PrNMI, selective cannabinoid receptor subtype blockers were administered, showing that PrNMI’s antiallodynic effects are mediated by CB1R activation [25]. Using different experimental paradigms, the non-psychoactive phytocannabinoid CBD as well as the psychoactive cannabis constituent THC both attenuated mechanical allodynia in mice treated with paclitaxel. Moreover, it is interesting that very low, ineffective doses of CBD and THC could be synergistic when given in combination. CBD also attenuated oxaliplatin- but not vincristine-induced mechanical allodynia, while THC significantly attenuated vincristine- but not oxaliplatin-induced mechanical allodynia. Once given together at low, ineffective doses, the combination significantly attenuated oxaliplatin- but not vincristine-induced mechanical allodynia [27]. In two different experiments, whether THC or CBD alone could attenuate or prevent cisplatin-induced tactile allodynia was also examined. In the first experiment, mice received repeated administrations of cisplatin to induce tactile allodynia; then, they received THC or CBD. In the second experiment, CBD or THC was given prior to each cisplatin administration. Cisplatin-induced tactile allodynia was attenuated by THC and CBD but not prevented by either cannabinoid [30]. Widening the interest to other cannabinoid-related pharmacological targets, Sierra et al. investigated the effect of paclitaxel administration and subsequent mechanical allodynia on CB1R and delta opioid receptor (DOR) heteromers. In their model, the Authors observed significant increases in CB1R-DOR heteromers in the DRG of mice with paclitaxel-induced CIPN. Then, they investigated the effect of the administration of subthreshold doses of a combination of ligands (CB1R agonist, Hu-210, and DOR agonist, SNC80), demonstrating that it was able to significantly attenuate allodynia in mice, while the administration of individual ligands was ineffective. Therefore, they concluded that CB1R-DOR heteromers upregulated during CIPN-associated mechanical allodynia could represent a potential druggable target for treatment of neuropathic pain in paclitaxel-induced CIPN [28]. The administration of neurotoxic drugs able to induce CIPN has also been associated with effects in the spinal cord, namely in the spinal cord dorsal horn (SCDH). Paclitaxel induces microglial activation and the production of proinflammatory mediators in the SCDH, which contribute to the development and maintenance of central sensitization and nocifensive behavior. Wu et al. tested in mice the hypothesis that activation of CB2R by M1-([3-benzyl-3-methyl-2,3-dihydro-1-benzofuran-6-yl]carbonyl) piperidine (MDA7), a highly selective CB2R agonist, modulates microglial dysregulation, suppresses the overexpression of brain-derived neurotrophic factor in SCDH microglia, and eventually attenuates animals’ nocifensive behavior. In this model, paclitaxel induced the expression of CB2R and production of interleukin (IL)-6 in microglia in the SCDH [29]. As evidenced by the examples previously summarized, cannabinoids can suppress neuropathic pain through activation of CB1R and/or CB2R receptors. However, unwanted CB1-mediated cannabimimetic effects can limit clinical use. To address this clinically relevant issue, Deng et al. tested if CP55,940 [(-)-3-[2-hydroxy-4-(1,1-dimethylheptyl)phenyl]-4-(3-hydroxypropyl)cyclohexanol], a potent cannabinoid that binds with similar affinity to CB1R and CB2R in vitro, was able to produce functionally separable CB1R- and CB2R-mediated pharmacological effects in vivo. To test this hypothesis, they selected a mouse model of toxic neuropathy produced by paclitaxel and they evaluated antiallodynic effects, possible tolerance, and cannabimimetic effects (e.g., catalepsy, hypothermia). The contribution of CB1R and CB2R to in vivo effects of CP55,940 was evaluated using CB1R knockout (KO), CB2RKO, and wild-type (WT) mice. Low-dose CP55,940 suppressed paclitaxel-induced allodynia in WT and CB2RKO mice, but not in CB1RKO mice. Low-dose CP55,940 also produced hypothermia in WT, but not CB1KO, mice. In WT mice, tolerance developed to CB1R-mediated hypothermic effects of CP55,940 earlier than to the antiallodynic effects. High-dose CP55,940 produced catalepsy in WT mice, which precluded determination of antiallodynic efficacy but provided sustained CB2R-mediated suppression of paclitaxel-induced allodynia in CB1KO mice. Interestingly, these antiallodynic effects were blocked by the CB2R antagonist 6-iodopravadoline (AM630). Taken together, specifically regarding in vivo peripheral effects, the results of this study indicate that CB1R and CB2R activations produce distinct suppression of neuropathic pain, and suggest the therapeutic potential of targeting the cannabinoid CB2R to avoid unwanted CNS effects associated with CB1R activation [31].

### Cannabinoid-Mediated Modulation of Neuroinflammation

Besides its capacity to produce analgesic effects, the endocannabinoid system can remarkably influence neuroinflammation, an event that is gaining increasing attention in the pathogenesis of CIPN [9, 37, 38]. This effect is due to the suppression of immune cell activation, proliferation and migration, and the activation of immune cell apoptosis. Administration of CP55,940 decreased the migration of rat macrophages through aCB1R- and CB2R- mediated mechanisms in both in vivo and in vitro models [39]. Furthermore, THC can indirectly inhibit the activation of T helper cells by suppressing antigen presentation in macrophages [40] and it inhibits the proliferation of human T cells stimulated with antigen-primed dendritic cells [41]. THC effects on the immune system are not simply related to its capacity to inhibit cell proliferation, since it induces apoptosis of mouse macrophages, T cells, and B cells in primary splenic and thymic cultures [42].

### Effect of Bortezomib Administration on the Cannabinoid System

This combination of antinociceptive and immunomodulatory effects makes very attractive the hypothesis that, through effective pharmacological intervention on the endocannabinoid system, CIPN might be treated, or even prevented. This hypothesis can be tested using suitable animal models.

Among the different forms of CIPN, bortezomib-induced peripheral neurotoxicity has the peculiar features of being very painful, with evidence of a remarkable importance of neuroinflammation in its pathophysiology, and to be reliably reproduced in rodent models. Here we briefly report the results of a study performed in a rat model of painful CIPN induced by long-term bortezomib administration [43–45] (detailed description of the Materials and Methods used in the study is available on the Bicocca Open Access Research Data website at http://dx.doi.org/10.17632/pb8dk5vkgv.1). Adult Wistar rats were treated with bortezomib (0.2 mg/kg i.v., 3 times/week for 8 weeks), a schedule that has been extensively investigated and it is known to induce the onset of peripheral neuropathy that reliably mimics the clinical features observed in patients receiving this drug to treat multiple myeloma [46]. Moreover, bortezomib administration according to this experimental paradigm induces increased expression of TRPV1, a non-canonical cannabinoid receptor, in DRG and SCDH [45].

**Fig. 1:**
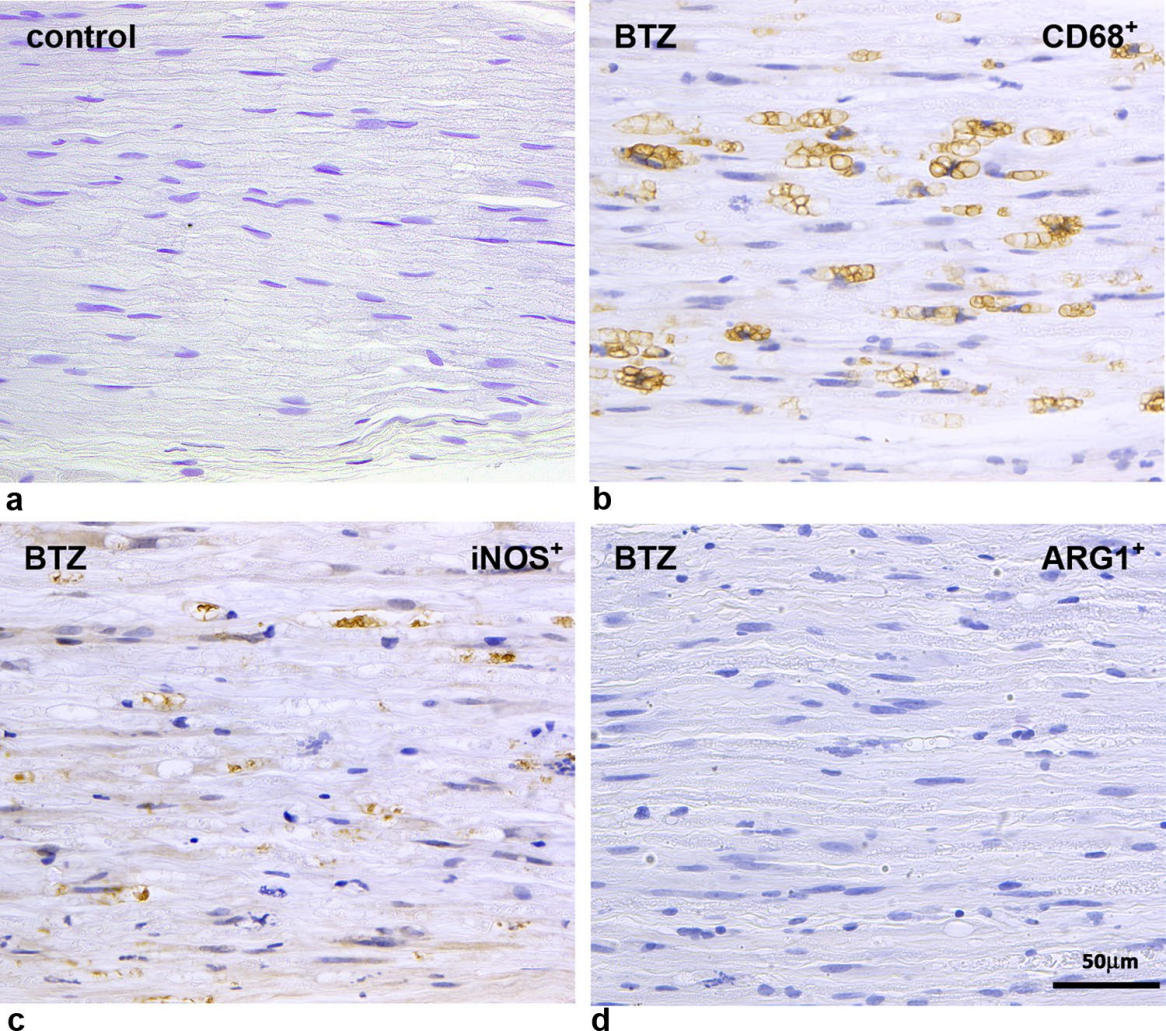
Representative images of macrophage infiltration in caudal nerves of a control and a bortezomib- (BTZ) treated rats taken from a previously published experiment [49] To investigate the macrophage infiltration immunohistochemistry was performed using anti-CD68 antibody to detect macrophage infiltrating cells (b) compared with control animals (a). In addition, anti-iNOS (inducible Nitric Oxide Synthase) antibody (c), and anti-ARG1 (Arginase-1) antibody (d) was used to discriminate M1 (proinflammatory) from M2 (anti-inflammatory) macrophages, respectively. While no infiltrating macrophages were observed in controls, marked M1 macrophage infiltration was present in the caudal nerves of BTZ-treated rats [49]

As previously mentioned, this model is also particularly interesting to investigate a possible role of cannabinoids in CIPN because it allows investigations to simultaneously address severe neuropathic pain and prominent neuroinflammation (Fig. 1). In this model, where conventional analgesics are scarcely effective and new investigational drugs have been tested [43, 47], immunomodulation using anti TNF-α antibodies [48], or repeated intravenous delivery of human immunoglobulins was able to significantly modify the disease course [49].

**Fig. 2:**
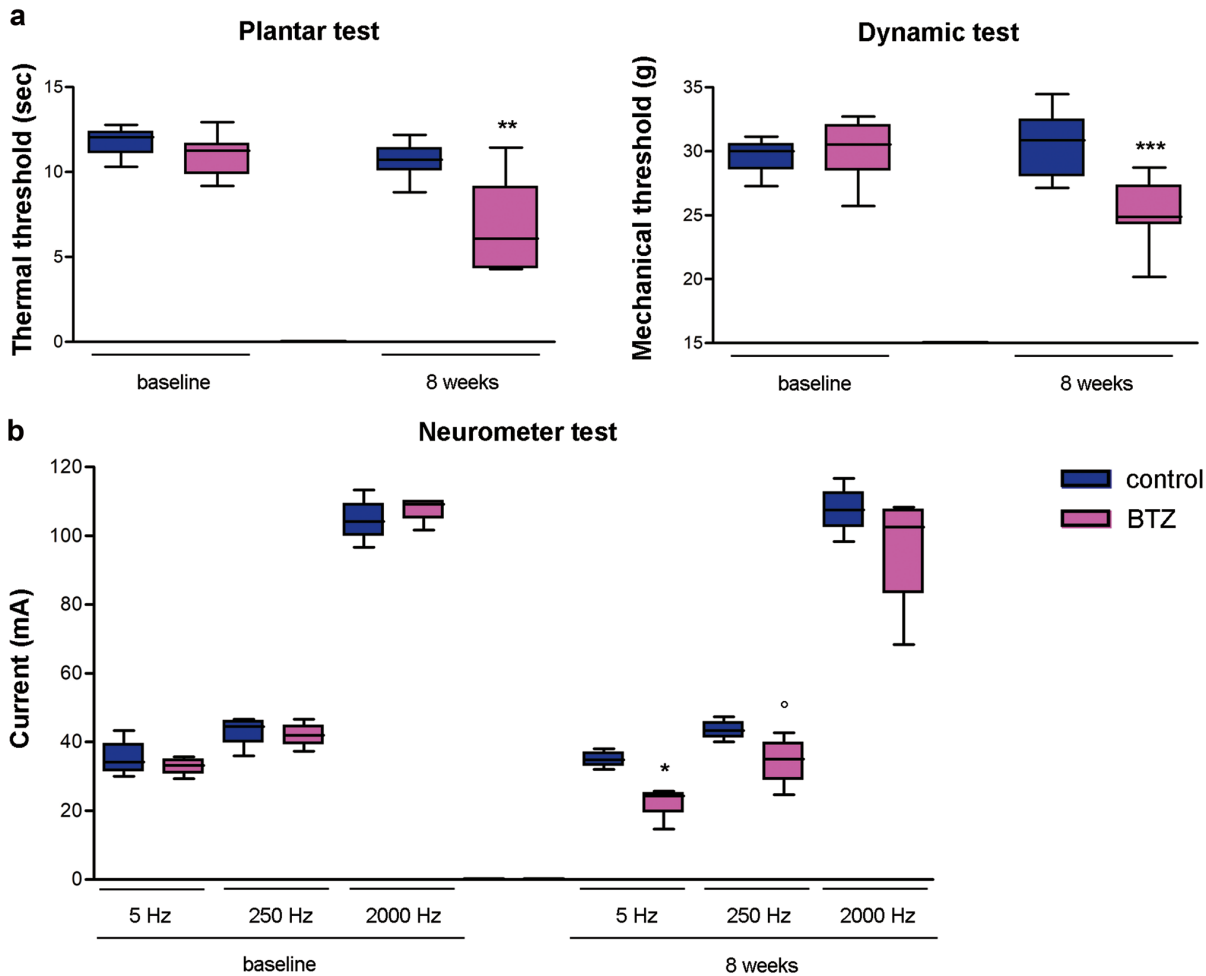
Animal model’s nocifensive behavior and current perception threshold (CPT) after bortezomib (BTZ) administration Withdrawal latency to an infrared heat stimulus was determined using a Plantar Test apparatus that showed thermal allodynia in BTZ-treated rats (a, left panel); mechanical threshold was assessed with the Dynamic Aesthesiometer Test device that showed mechanical allodynia in BTZ-treated rats (a, right panel), b) the Neurometer device was used to evaluate the CPT as a quantitative measure of nerve function by selectively depolarizing different subpopulations of afferent fibers. BTZ treatment significantly increased the sensitivity of the A-delta and C fibers function, which resulted in a behavioral response to a lower current stimulus than the control group, while BTZ had no effect on large myelinated fibers (b). °P<0.05 vs control 250Hz; *P<0.05 vs control 5 Hz; **P<0.01 vs control; ***P<0.001 vs control. For more details about Materials and Methods, see Supplementary material

After bortezomib administration, rats showed thermal and mechanical allodynia (Fig. 2a) due to altered functioning of small myelinated and unmyelinated fibers, based on neurophysiological stimulation of peripheral nerves at different frequencies (Fig. 2b).

**Fig. 3:**
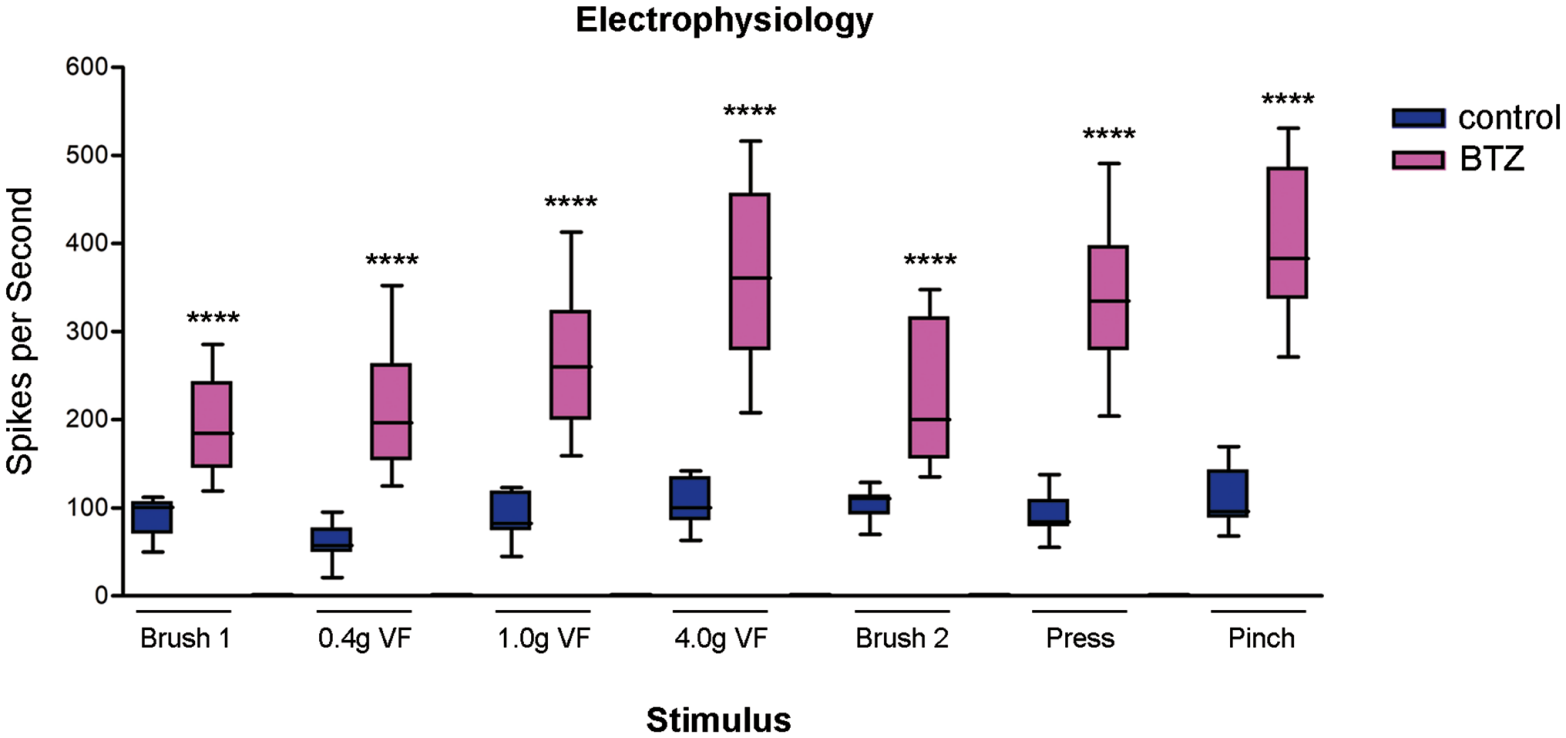
Extracellular electrophysiological recording in the SCDH of bortezomib (BTZ)-treated rats BTZ-treated animals showed significant wide dynamic range neurons (WDRN) hyperexcitability during all evoked response by light tactile (sable-hair brush, light Von Frey (VF) hairs), moderate noxious tactile (press) and painful stimuli (pinching). Control, bortezomib (BTZ) ****P<0.0001 vs control. For more details about Materials and Methods, see Supplementary material

Moreover, the animals’ nocifensive behavior was paralleled by increased SCDH wide dynamic range neurons excitability, a common feature in neuropathic pain already reported and described in detail in CIPN animal models [50, 51] (Fig. 3).

**Fig. 4:**
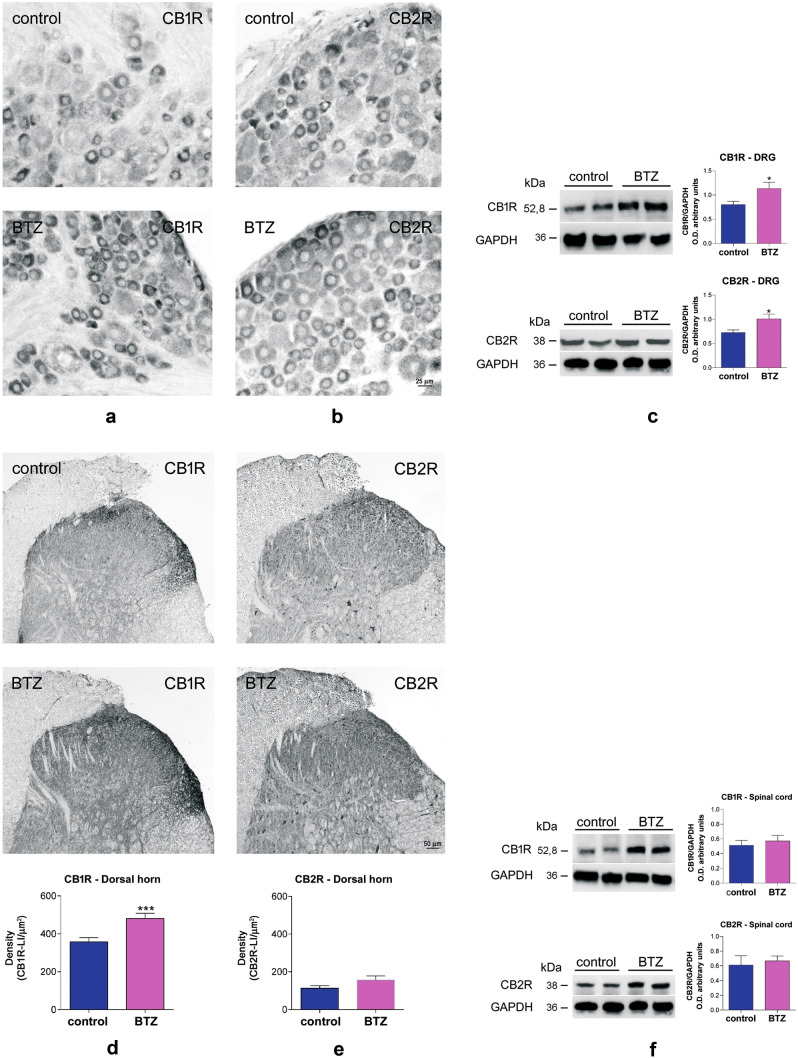
Effects of bortezomib (BTZ) treatment on CB1R and CBR2 expression in DRG and SCDH Localization of CB1R-like immunoreactivity (LI) in the DRG of a control (a, upper panel) and a BTZ-treated rat (a, lower panel); localization of CB2R-LI in the DRG of a control (b, upper panel) and a BTZ-treated rat (b, lower panel); western blot and results quantification comparing control vs BTZ-treated animals (c). Localization of CB1R-L5 in the SCDH of a control (d, upper panel) and a BTZ-treated rat (d, intermediate panel) with optical density quantification (d, lower panel); localization of CB2R-LI in the SCDH of a control (e, upper panel) and a BTZ-treated rat (e, intermediate panel), with optical density quantification (e, lower panel); western blot and results quantification of the whole spinal cord comparing control vs BTZ-treated animals (f). *P<0.05 vs control; ***P<0.001 vs control. For more details about Materials and Methods, see Supplementary material

In this well-characterized model, the effects of bortezomib treatment on CB1R and CB2R expression and distribution in the DRG and SCDH cord were investigated. The immunohistochemical distribution of CB1R and CB2R indicated, after direct counting of 8000–10,000 neurons in each group performed by a blinded examiner, that bortezomib administration induces an increase in the number of CB1R- and CB2R-positive DRG neurons in comparison to untreated controls (34.3% vs 26.8% for CB1R and 28.7% vs 21.3% for CB2R, *p* < 0.001 in both cases, Fig. 4a, b). Morphometric analysis, after direct measuring of about 2500 neurons in each group, showed that CB1R- and CB2R-positive neurons fell in the size range of 7–47 µm (22 µm mean diameter) and 8–63 µm (27 µm mean diameter), respectively, and allowing to classify them as nociceptors. Western blot analysis evidenced that the differences in the percentage of CB1R- and CB2R- positive DRG neurons resulted in a significant increase in CB1R and CB2R expression (Fig. 4c).

The densitometry analysis performed on the SCDH showed a mild albeit significant increase in CB1R immunoreactivity in bortezomib-treated rats vs controls (Fig. 4d), while no significant difference was found for CB2R (Fig. 4e). Western blot analysis performed on the whole spinal cord did not show any significant difference in CB1R or CB2R expression between the two groups of animals (Fig. 4f).

While future studies are needed to gain insight into the possible interaction of CBRs and TRPV1 in bortezomib-induced peripheral neuropathy, taking into account that TRPV1 sensitization is one of the aspects of bortezomib-induced neurotoxicity [45], it is interesting that CB2R has been found to colocalize with TRPV1 in avulsed human DRG neurons [52] and that CB2R agonists diminish TRPV1 activation by depleting cAMP levels [52, 53].

## Cannabinoids in the Treatment of CIPN

Interest in exploring the possibility to administer cannabinoids in CIPN patients was initially evoked by the results of a small pilot study in 2014, where the authors recommended further investigation of the effects of the cannabinoid agent nabiximols against CIPN in large, randomized, placebo-controlled trials [54]. That study reported the results of a randomized, placebo-controlled crossover pilot trial done in 16 patients with established chemotherapy-induced neuropathic pain. When examining the whole group, there was no statistically significant difference between the treatment and the placebo groups on the 0–10-point numeric rating scale for pain intensity (NRS-PI). However, a responder analysis demonstrated that there were five participants who reported a 2-point or greater reduction in pain that trended toward statistical significance and the number needed to treat was 5.

Despite growing evidence in the literature, the question of the role of cannabinoids in CIPN still needs to be answered. Therefore, the presence of 4 clinical trials in the list of those currently registered in ClinicalTrials.gov might represent a critical turning point in the attempt to clarify this issue. However, these clinical trials have fairly different aims and designs.

The aims of the first study (“The Kinetics of Endocannabinoids in Patients With Chemotherapy Induced Peripheral Neuropathy by Using Medical Cannabis,” ClinicalTrials.gov Identifier: NCT04376437) are to evaluate the changes in level of endocannabinoids following continuous exposure to phytocannabinoids and the long-term effect of medical cannabis on CIPN (medical cannabis is indicated in Israel, where the study will be conducted, for the treatment of chronic pain, spasticity and for the control of pain and other symptoms in patients with cancer). This is a small (40 patients), single group, open label clinical trial. All patients, aged 18–80, will start with 250 mcg cannabis flos (Syqe Medical Cannabis inhaler) BID and will follow the titration plan of dose modification according to CIPN symptom relief and the occurrence of adverse events. A maximum dose of 2000 mcg per day will be reached at the end of titration period, which is continuous for 15 days. At the 10-week visit, all patients will be discontinued from the treatment. In case of worsening of neuropathy at any point during the 4 weeks of follow-up, patients will be allowed to restart with inhaled medical cannabis treatment for no more than 4 weeks. The primary outcome measure of this clinical trial will be the measurement of the changes in the level of 150 different endocannabinoids measured in blood samples collected during the 4 months of participation in the study. However, changes from baseline in neuropathic pain and in quality of life using the Functional Assessment of Cancer Therapy – Gynecologic Oncology Group-Neurotoxicity (FACT-GOG-Ntx) questionnaire and the Brief Pain Inventory (BPI) tool will also be assessed. To avoid pharmacological interaction, patients are not eligible if they used cannabis or synthetic cannabinoids in the last 2 weeks prior to enrollment, or if they use alcohol, barbiturates, opiates, primidone, carbamazepine, rifampin, rifabutin, troglitazone, or hypericum perforatum.

The second study (“Cannabidiol for Prevention of Chemotherapy-induced Peripheral Neuropathy (CINCAN-2), NCT04582591”) is also an open label, single arm clinical trial of the same size (40 patients, aged ≥ 18) aimed at the assessment of the preventive effect of CBD against CIPN. The primary outcome measure for efficacy for patients receiving paclitaxel-based chemotherapy is the difference in baseline vibrograms of patients treated with CBD compared to vibrograms at follow-up 3 months after the end of the 6th course of chemotherapy or the last course of chemotherapy (if before course no. 6). For patients receiving oxaliplatin-based chemotherapy, baseline vibrograms of patients treated with CBD will be compared with vibrograms at follow-up 3 months after the end of the 4th course of chemotherapy or the last course of chemotherapy (if before course no. 4). Specific and validated questionnaires will be used as secondary outcome measures to assess the effects of CBD on quality of life and CIPN (i.e., the European Organization for Research and Treatment of Cancer (EORTC) Quality of Life Questionnaire, EORTC-QLQ-C30, and the EORTC Chemotherapy-Induced Peripheral Neuropathy Questionnaire Module, EORTC-QLQ-CIPN20) Exclusion criteria include use of cannabinoids in the last 4 days prior to enrollment and the use of clobazam, while antidepressants and antiepileptic drugs are allowed if their dosage was stable in the last 30 days.

These two studies are unlikely to provide conclusive evidence in favor or against a clinically relevant role of cannabinoids in CIPN, mostly because of their open-label design and small size.

However, two other larger studies are already recruiting patients, and they not only have a more rigorous design, but also selected a more robust and reliable panel of outcome measures.

The “Cannabinoids for Taxane Induced Peripheral Neuropathy” study (NCT03782402) is a 100-patient randomized, parallel assignment, triple blinded (participant, investigator, and outcome assessor) phase 2 clinical trial. The primary outcome measures include assessments using the BPI-Short Form for pain severity and the BPI pain interference subscale for functional impairment. The study outcomes also include secondary measures of sensory perception using the FACT-GOG-Ntx and the Total Neuropathy Score, clinical version (TNSc). Women (aged 21–60) with an Eastern Cooperative Oncology Group (ECOG) performance status of 2 (i.e., ambulatory and capable of all self-care but unable to carry out any work activities) or 3 (capable of only limited self-care, confined to bed or chair more than 50% of waking hours) receive cannabinoids with different concentrations of THC and CBD (or placebo) to treat CIPN following paclitaxel- or docetaxel-based chemotherapy for breast cancer. No restriction of the use of other drugs is planned, except for warfarin.

Finally, the “Effect of Hemp-CBD on Patients With CIPN (Coala-T-CBD)” study (NCT04398446) has the aim to assess the effect of a hemp-based CBD product, Ananda Hemp Spectrum Gelcaps, on the severity and duration of CIPN after therapy that included taxanes or oxaliplatin. In this randomized, parallel triple blinded (participant, investigator, and outcome assessor) phase 2 clinical trial, the primary outcome measures are physician-assessed change in pressure/touch and vibration sensation during treatment and at follow-up, change in EORTC-QLQ-C30, EORTC-QLQ-CIPN20, BPI-Short Form, and in Patient-Reported Outcomes Measurement Information System (PROMIS) Sleep Disturbance Questionnaire. Patients are included if they are aged ≥ 21 and developed CIPN (National Cancer Institute Common Toxicity Criteria sensory grade ≥ 2, motor grade < 2) and they receive hemp-based CBD, 3 daily dosing for 12 weeks, or placebo. Due to potential CBD-drug interaction, patients are not eligible if they receive any opioids, erythromycin, clarithromycin, fluconazole, itraconazole, sulfamethoxazole, clopidogrel, rifampin, warfarin, antiepileptic agents (including phenytoin, carbamazepine, valproic acid, but excepting of gabapentin, clonazepam or diazepam). Routine use of cannabis products for medicinal or recreational purposes (defined as > 4 times/month) or of any illicit drug precludes inclusion in the study.

## Exercise and Endocannabinoids

A number of studies provide some indication that exercise may treat or prevent CIPN [55], and their results suggest that more severe CIPN symptoms tend to occur in patients who are older, less aerobically fit, and overweight or obese. Up to now, 7 clinical trials have been registered in ClinicalTrials.gov, and 5 of them are already actively recruiting patients with different types of CIPN. Despite emerging evidence for at least some level of efficacy, the mechanism(s) through which physical therapy, and exercise in general, could exert their beneficial effects remain unclear [56, 57].

However, under this mechanistic perspective, it is potentially interesting that exercise and endocannabinoid release seem to be strongly related [28], thus creating a theoretical link between the two investigated treatments for CIPN. In fact, the hypothesis that circulating endocannabinoids coordinate a system-wide response to seek, consume, and store energy suggests that increased energy utilization results in higher circulating levels of endocannabinoids to replenish energy stores, and it is possible that the increased amount of endocannabinoids in the circulation following exercise come from skeletal muscle. In support of this hypothesis, it has been demonstrated that 30–90 min of moderate exercise increases circulating concentrations of AEA, a fatty acid neurotransmitter acting on CB1R in the CNS, and CB2R in the periphery [58, 59]. However, it is possible that differences can exist in different conditions, for example in women vs men. In fact, a study performed in women showed that moderate-vigorous physical activity measured over 6 days is positively correlated with circulating AEA concentrations [60], while no differences in basal concentrations of AEA and 2-AG were found between active and sedentary normal weight men [61]. Overall, though the results are generally consistent regarding AEA across the different studies, this is not the case for 2-AG, another endocannabinoid able to bind to both CB1R and CB2R [58, 59, 62, 63].

## Conclusion

CIPN management is now one of the emerging critical issues in cancer treatment, and the possibility that its signs and symptoms become permanent poses a potentially huge burden of additional morbidity on cancer survivors. Therefore, the search for effective and reliable new therapeutic strategies is extremely important and any potential targets deserve to be explored. However, from the review of the currently ongoing registered clinical trials, as well as from other reviews based on published and unpublished results [3], it seems that non-pharmacological treatments are now the leading option under investigation. In most cases, these non-pharmacological studies are not supported by a strong rationale, but their diffusion represents the tangible evidence of the failure in identifying druggable targets to modify the severity and course of CIPN.

On the other hand, the endocannabinoid system has been investigated only partially to this aim, sometimes with animal models that are not able to fully recapitulate the human condition. Moreover, it is unlikely that the clinical trials described above will be able to offer a final answer on the potential efficacy of cannabinoids in CIPN patients. However, particularly the 2 largest phase 2 studies are properly designed to provide useful information that will allow, if positive, to eventually design an evidence-based phase 3 study. To this aim, we believe that the ACTTION recommendations on trial designs for CIPN prevention might provide very helpful guidelines [64].

## Supplementary Information

Below is the link to the electronic supplementary material.

Supplementary file1 (PDF 525 kb)

Supplementary file2 (PDF 508 kb)

Supplementary file3 (PDF 462 kb)

Supplementary file4 (PDF 462 kb)

Supplementary file5 (PDF 508 kb)

Supplementary file6 (PDF 508 kb)

Supplementary file7 (PDF 463 kb)

Supplementary file8 (DOCX 55 kb)

Supplementary file9 (TIF 108 kb)
