# Supplementary material for: Cannabinoids: an Effective Treatment for Chemotherapy-Induced Peripheral Neurotoxicity?
Source: Neurotherapeutics. 2021 Oct 19;18(4):2324–36. doi: 10.1007/s13311-021-01127-1 (PMC8804126; doi:10.1007/s13311-021-01127-1)
Supplement: Supplementary file 8 — Supplementary file8 (DOCX 55 kb) [file 13311_2021_1127_MOESM8_ESM.docx]

**Cannabinoids: an effective treatment for chemotherapy-induced peripheral neurotoxicity?**

Guido Cavaletti *‡, Paola Marmiroli †‡, Cynthia L. Renn §, Susan G. Dorsey§, Maria Pina Serra ¶, Marina Quartu ¶, Cristina Meregalli *‡

* Experimental Neurology Unit, School of Medicine and Surgery, University of Milano Bicocca,

via Cadore 48, Monza, Italy

† Department of Biotechnology and Biosciences, University of Milano-Bicocca, Piazza della Scienza, 2, Milano, Italy

‡ Milan Center for Neuroscience, University of Milano Bicocca, Piazza Ateneo Nuovo 1, Milano, Italy

§ Department of Pain and Translational Science, School of Nursing, University of Maryland. 655 West Lombard Street, Baltimore, MD 21201 USA

¶ Department of Biomedical Sciences, University of Cagliari, Cittadella Universitaria, 09042 Monserrato, Italy

**Materials and Methods of the bortezomib-induced peripheral neurotoxicity study**

Animals and Ethics Statements

A total of 20 adult female Wistar rats (Envigo, Udine Italy), aged 10-11 weeks at the beginning of the experiment, were used for the **experiment 1** at UNIMIB (University of Milan Bicocca, Milan), and a total of 32 adult female Wistar rats (Charles River Laboratories, Frederick, MD, USA), aged 10-11 weeks at the beginning of the experiment, were used for the **experiment 2** at UMB (University of Maryland, Baltimore). Animals were housed in a limited access animal facility with controlled room temperature (22 ± 2°C) and relative humidity (55 ± 10%) and under an artificial 12 h light/dark cycle (light 7 a.m.–7 p.m.). The care and husbandry of the animals were in conformity with the institutional guidelines in compliance with Italian legislation (D.L n. 26/2014). The Institutional Animal Care and Use Committee of the University of Maryland School of Medicine approved the study at UMB while the experimental plan at UNIMIB was examined and approved by the Ethics Committee of the University of Milano-Bicocca and Italian Ministry of Health (approval number # 842/2018-PR).

Drug Administration and Euthanasia

Bortezomib (BTZ) (LC Laboratories, Woburn, MA) was dissolved in 10% Tween 80, 10% ethanol, and 80% sterile saline. A total of 26 rats was treated with BTZ for 8-week period by tail vein injection (0.20 mg/kg/day three times/week; see [1], the remaining animals were left untreated and used as controls. The experimental plan (summarized in Table 1) was divided into two parts: **experiment 1** was carried out at UNIMIB to characterize the BTZ-induced painful peripheral neuropathy in Wistar rats; **experiment 2** was performed at UMB to investigate the alterations of the wide dynamic range neurons excitability and the quantitative measure of nerve function by neurometer after BTZ treatment. All tests were operated by an experimenter blinded to groupings and in the controlled test room. At the end of all experiments, rats were euthanized by CO_2_ inhalation followed by cervical dislocation and partially used for biological sampling.

Assessment of mechanical allodynia

Mechanical tests were performed by a single experimenter who was blind to the treatment groups to determine the presence of chemotherapy-induced allodynia as previously described [2]. Rats were placed into a plexiglass cage positioned on an elevated wire mesh floor and were allowed to adapt to the testing environment for at least 10 min. Tactile hind paw withdrawal threshold in rats was automatically recorded. Three thresholds were taken for each paw. The electronic Von Frey apparatus progressively increases puncture pressure to the plantar surface, reaching up to 50 grams within 20 seconds. An upper limit cutoff of 20 s was fixed.

Assessment of paw withdrawal latencies to heat stimulation

Plantar tests were performed by a single experimenter who was blind to the treatment groups to determine the response to thermal stimuli as previously described [3]. Animals were placed into a plexiglas cage on an elevated glass floor for a 10 min acclimatization period followed by testing. Radiant heat was located under the glass platform and was positioned under the centre of the rear paw of rats, and withdrawal time was recorded as withdrawal latency (sec). The intensity of the heat source was fixed to an infra-red setting of 40W. The mean of the four readings was used for data analysis.

Current Perception Threshold (CPT) Measurements

For the current perception threshold (CPT) measurement in the footpad, the rats were placed in a restraint jacket and suspended from a frame 3 inches above the bench surface with the paws dangling freely [4]. Anesthesia was induced in a chamber with 3% isoflurane carried in oxygen followed by 0.75-1% titrate isoflurane by nose cone for maintenance throughout the procedure, to allow a withdrawal response of the hind paw to occur without the rat struggling to escape from the restraint. The stimulus electrode was applied to the plantar surface of the left hind paw and the ground electrode was applied to the left ankle. Using the Neurometer (Neurotron Inc., Baltimore, MD), sine-wave transcutaneous electrical stimuli were applied at three frequencies: 250 Hz activating A-δ fibers and 5 Hz activating C-fibers involved in pain perception, while 2000 Hz activates large myelinated fibers, with the current increasing stepwise until a nocifensive response (paw withdrawal or flick) was observed. When a response was elicited, the stimulus was stopped and the amount of current (µA) delivered at the time of the response was recorded [5]. A nocifensive response was defined as a brisk withdrawal or flicking of the hind paw. The hind paw was tested three times at each frequency and the current perception threshold was the average of those three trials.

Wide Dynamic Range (WDR) neurons recordings

Extracellular electrophysiological recording was performed to measure the activity of wide dynamic range neurons (WDRN) in the spinal cord dorsal horn. Anesthesia was induced and maintained similarly to CPT measurement analysis, and prior to the laminectomy surgery for the spinal cord electrophysiological recordings, rats were intraperitoneally injected with Pentobarbital 4 mg/kg. The animals underwent laminectomy to expose the L4-L5 spinal segment and a tungsten microelectrode (10 μm-tip, Frederick Haer Co., Brunswick, ME, USA) was vertically positioned with an electronical micropositioner at 400–600 μm in depth in the spinal cord dorsal horn (Model 660 micropositioner, David Kopf Instruments, Tujunga, CA, USA). SciWorks (Datawave Technologies, Loveland, CO, USA) was used to acquire and digitalize the neuronal electrical activity during spontaneous and evoked response by light tactile (sable-hair brush, light Von Frey (VF) hairs), moderate noxious tactile (press) and painful stimuli (pinching). Neuronal activity was discriminated, sorted and analyzed by principal components analysis offline using SciWorks (v7.0, Datawave Technologies, Berthoud, CO, USA). Stimulus-evoked activity was quantified by calculating the number of spikes/seconds [6, 7].

Sampling

Immediately after sacrifice, the L4-L6 DRGs with the corresponding spinal cord segments were rapidly dissected out and either frozen at −80°C for western blot analyses or fixed by immersion in freshly prepared 4% phosphate-buffered paraformaldehyde, pH 7.3, for 4–6 h at 4°C, and then rinsed overnight in 0.1 M phosphate buffer (PB), pH 7.3, containing 20% sucrose for immunohistochemistry. After sucrose infiltration, samples were embedded in Optimal Cutting Temperature (OCT) medium for cryostat sectioning. For each assay, the investigator was blind with respect to the experimental condition of rats.

Western blot

Tissue homogenates of 5 pools of 3 DRGs (L4-L6) and 5 spinal cord segments from 5 animals per group (5 BTZ-treated and 5 controls) were homogenized in distilled water containing 2% sodium dodecyl sulfate (SDS; 300 μl/100 mg of tissue) and a cocktail of protease inhibitors (completeTM, Mini Protease Inhibitor Cocktail Tablets, Cat# 11697498001, Roche, Basel, Switzerland).

Protein concentrations were determined using the Lowry method [8] with bovine serum albumin as the standard. Proteins, 40 μg for each tissue homogenate, diluted 3:1 in 4× loading buffer (NuPAGE LDS Sample Buffer 4×, Novex ThermoFisher Scientific, Waltham, MA, USA), were heated to 95°C for 7 min and separated by SDS-polyacrylamide gel electrophoresis using precast polyacrylamide gradient gel (NuPAGE 4–12% Bis-Tris Gel Midi, Novex, ThermoFisher Scientific, Waltham, MA, USA) in the XCell4 Sure-Lock Midi-Cell chamber (ThermoFisher Scientific, Waltham, MA, USA). Internal molecular weight (MW) standards (Precision Plus Protein WesternC Standards, Bio-Rad, Hercules, CA, USA) were run in parallel. Two gels at a time were run for Coomassie staining and immunoblotting, respectively. Proteins for immunoblotting were electrophoretically transferred on a polyvinylidene fluoride membrane (Amersham Hybond-P, GE Healthcare, Little Chalfont, UK) using the Criterion Blotter (Bio-Rad). Blots were blocked by immersion in 20 mM Tris base and 137 mM sodium chloride (TBS), containing 0.1% Tween 20 (TBS/T) and 5% milk powder, for 60 min, at room temperature. The primary antibodies were rabbit polyclonal antibodies directed against CB1R (Synaptic System, Göttingen, Germany), diluted 1:500, and CB2R (Cayman Chemical, Ann Arbor, Mi, USA), diluted 1:1000 in TBS/T containing 5% milk powder and 0.02% sodium azide. Incubations with primary antiserum were carried out for one night at 4°C. After rinsing in TBS/T, blots were incubated at room temperature, for 60 min, with peroxidase-conjugated goat anti-rabbit serum (Cat#9169, Sigma Aldrich, St. Louis, MO, USA), diluted 1:10,000 in TBS/T. Controls for equal-loading of the wells were obtained by immunostaining the membranes, as above, using a mouse monoclonal antibody against glyceraldehyde-3-phosphate dehydrogenase (GAPDH; MAB374, Merck Millipore), diluted 1:1,000, as the primary antiserum, and a peroxidase-conjugated goat anti-mouse serum (AP124P, Merck Millipore), diluted 1:5,000, as the secondary antiserum. To control for non-specific staining, blots were stripped and incubated with the relevant secondary antiserum. After rinsing in TBS/T, protein bands were developed using the Clarity Max ECL Substrate (Cat# 1705062, Bio-Rad), according to the protocol provided by the manufacturer, and visualized using the ImageQuant LAS-4000 (GE Healthcare). Approximate MW and relative optical density (O.D.) of the labeled protein bands were evaluated by an examiner who was not aware of the rat line from which the tissue analyzed was obtained. The ratio of the intensity of the CB1R- and CB2R-positive bands to the intensity of the GAPDH-positive ones was used to compare the relative expression levels of these proteins in the controls and BTZ-treated rats.

The O.D. was quantified by Image Studio Lite Software (Li-Cor, [http:/www.licor.com/bio/products/software/image_studio_lite/](http://www.licor.com/bio/products/software/image_studio_lite/)) and is shown as histograms in Figure 4.

Immunohistochemistry

Cryostat sections (14 μm thick) of 3 DRGs (L4-L6)/animal (n=3) and lumbar-sacral spinal cord segments (n=5) for each group (control and BTZ-treated) were collected on chrome alum-gelatin coated slides and processed by the avidin–biotin–peroxidase complex (ABC) immunohistochemical technique. The endogenous peroxidase activity was blocked with 0.1% phenylhydrazine in phosphate buffered saline (PBS) containing 0.2% Triton X-100 (PBS/T) followed by incubation with 20% of either normal goat or normal horse serum (Vector, Burlingame, CA, USA) for 1 h at RT and then incubated with rabbit polyclonal antibody against CB1R (Synaptic System, Germany), diluted 1:1000, and against CB2R (Cayman Chemical, Ann Arbor, Mi, USA), diluted 1:600 in PBS/T, as primary antibodies. Incubations with primary antiserum were carried out overnight at 4 °C. Biotin-conjugated goat anti-rabbit serum (Vector, Burlingame, CA, USA), diluted 1:400, was used as secondary antiserum. The ABC (BioSpa Div. Milan, Italy), diluted 1:250, followed by a solution of 0.1 M PB, pH 7.3, containing 0.05% 3,3′-diaminobenzidine (Sigma, Milan, Italy), 0.01% hydrogen peroxide and 0.04% nickel ammonium sulfate was used to reveal the reaction product. Incubations with secondary antiserum and ABC lasted 60 min and were performed at RT. Negative control preparations were obtained by omitting the primary antibody or by substituting it with normal goat serum. Slides were examined by the same examiner blinded to animals’ treatment with an Olympus BX61 microscope and digital images were captured with a Leica DF 450C camera.

Image Densitometry

For the quantitative evaluation of the CB1R and CB2R immunohistochemical labeling, representative 10× magnification microscopic fields, were taken from 15 DRG sections (5 L4, 5 L5, 5 L6)/animal and five transverse sections of spinal cord dorsal horn/animal for each condition. The sections were blindly analyzed with ImageJ (<http://rsb.info.nih.gov/ij/>) to calculate the density of immunoreactivity per µm^2^. To exclude the background staining, mean gray values from the unstained areas were subtracted from the gray values of the immunostained regions.

Statistical Analyses

The data from behavioral test, Neurometer and WDR neurons recordings were analyzed by nonparametric unpaired two-tailed Mann-Whitney test, and WB and immunohistochemical data were statistically evaluated using the unpaired, two-sided Student’s *t* test. Statistical analyses were all carried out with the PRISM, GraphPad 6 Software (San Diego, CA, USA), with the significance level set at *p* < 0.05.


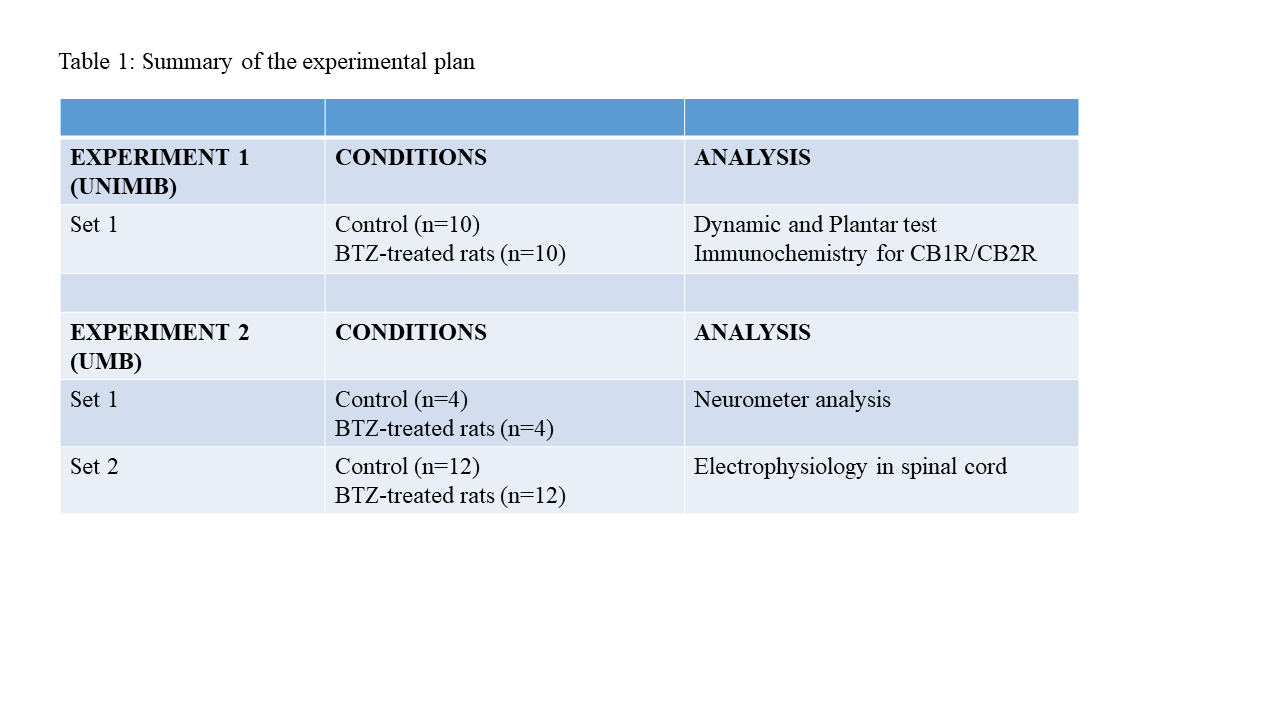


Table 1: Summary of the experimental plan. The experimental plan was composed of two experiments. In **experiment 1**, one set of rats were employed for the behavioral and immunohistochemical analyses. Here, the animals were randomized into 2 groups, one treated with bortezomib (BTZ) and one left untreated (control). In **experiment 2**, the animals were randomized into 2 groups similarly to experiment 1 (BTZ and control). Here, one set of rats was dedicated to neurometer analysis and the second one for electrophysiological analysis in the dorsal horn of the spinal cord.

Reference:

[1] Meregalli C, Canta A, Carozzi VA, Chiorazzi A, Oggioni N, Gilardini A, et al. Bortezomib-induced painful neuropathy in rats: a behavioral, neurophysiological and pathological study in rats. Eur J Pain 2010;14:343-350.

[2] Meregalli C, Monza L, Chiorazzi A, Scali C, Guarnieri C, Fumagalli G, et al. Human Intravenous Immunoglobulin Alleviates Neuropathic Symptoms in a Rat Model of Paclitaxel-Induced Peripheral Neurotoxicity. Int J Mol Sci 2021;22:1058.

[3] Quartu M, Carozzi VA, Dorsey SG, Serra MP, Poddighe L, Picci C, et al. [Bortezomib Treatment Produces Nocifensive Behavior and Changes in the Expression of TRPV1, CGRP, and Substance P in the Rat DRG, Spinal Cord, and Sciatic Nerve](https://www.ncbi.nlm.nih.gov/pmc/articles/PMC4022313/). Biomed Res Int 2014;2014:180428.

[4] Ueda H, Matsumoto M. Novel sensory fiber-specific nociception test with the Neurometer. Nihon YakurigakuZasshi 2008;131:367-371.

[5] Matsumoto M, Inoue M, Hald A, Yamaguchi A, Ueda H. Characterization of three different sensory fibers by use of neonatal capsaicin treatment, spinal antagonism and a novel electrical stimulation-induced paw flexion test. Mol Pain 2006;2:16.

[6] Renn CL, Carozzi VA, Rhee P, Gallop D, Dorsey SG, Cavaletti G. Multimodal assessment of painful peripheral neuropathy induced by chronic oxaliplatin-based chemotherapy in mice. Mol Pain 2011;7:29.

[7] Marmiroli P, Riva B, Pozzi E, Ballarini E, Lim D, Chiorazzi A, et al. Susceptibility of different mouse strains to oxaliplatin peripheral neurotoxicity: Phenotypic and genotypic insights. PLoS One 2017;12(10):e0186250.

[8] Lowry OH, Rosebrough NJ, Farr AL, Randall RJ. Protein measurements with the Folin phenol reagent. J Biol Chem 1951;193:265–275.
