# Supplementary figures and images for: Cannabinoids: an Effective Treatment for Chemotherapy-Induced Peripheral Neurotoxicity?
Source: Neurotherapeutics. 2021 Oct 19;18(4):2324–36. doi: 10.1007/s13311-021-01127-1 (PMC8804126; doi:10.1007/s13311-021-01127-1)

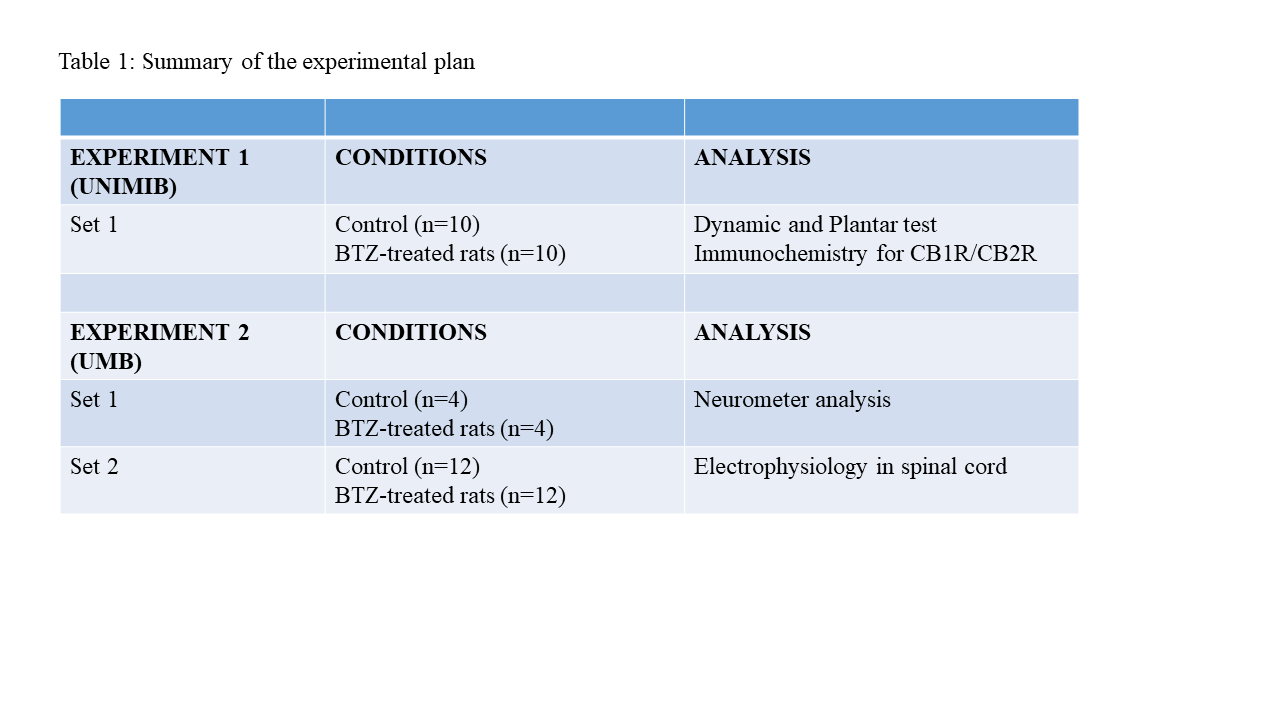

Supplement: Supplementary file 9 — Supplementary file9 (TIF 108 kb) [file 13311_2021_1127_MOESM9_ESM.tif]
